# L2 students’ academic emotions in teacher feedback and GAI-generated feedback on argumentative writing

**DOI:** 10.3389/fpsyg.2026.1816731

**Published:** 2026-06-11

**Authors:** Lian Wang, Mei An

**Affiliations:** School of Foreign Languages, Guizhou University of Finance and Economics, Guiyang, Guizhou, China

**Keywords:** academic emotions, argumentative writing, emotion-regulation strategies, GAI-generated feedback, teacher feedback

## Abstract

With the rise of generative artificial intelligence (GAI) in the field of second (foreign) language writing, GAI-generated feedback has garnered increasing scholarly attention. While previous research has compared the efficacy of GAI and teacher feedback, the academic emotions elicited by GAI during the revision process remain under-explored. To fill this lacuna, this study employed a mixed-methods research design to investigate the emotional experiences and corresponding regulation strategies of L2 students in both teacher and GAI-generated feedback contexts. Eighty-six Chinese master’s students participated in the study, with qualitative data gathered from written retrospective reflections, essays with teacher feedback, screenshots documenting the GAI-assisted revision process, and semi-structured interviews. The results revealed that students experienced higher levels of positive emotions in teacher-feedback contexts, whereas GAI-generated feedback was associated with more negative emotions. Furthermore, the findings identified distinct occurrences, types, and developmental trajectories of emotions across the two feedback conditions. This study offers important pedagogical implications, encouraging teachers to adopt hybrid feedback strategies and suggesting ways to help students manage negative emotions by fostering a growth mindset and enhancing feedback literacy.

## 1 Introduction

Second language (L2) writing is an emotionally laden process, writers navigating the feedback process inevitably experience a spectrum of complex emotions (Han and Hyland, 2019; Yu et al., 2021; Geng and Yu, 2024). Feedback, a critical tool in L2 writing assessment, refers to the written or oral comments and revision suggestions provided on the language and content of a learner’s work (Liu and Xin, 2025). As an essential component of writing instruction, feedback is widely recognized as a valuable means of scaffolding L2 development and effectively enhancing the quality of student writing output. A growing body of research indicates that L2 writers act as proactive agents rather than passive recipients during this process, with their emotions significantly influencing how they engage with the feedback they receive (Pitt and Norton, 2016; Papi et al., 2020; Li and Zhang, 2026). Given this perspective, it is crucial to account for L2 students’ academic emotions within the feedback cycle. Current literature regarding student emotions in writing revision varies significantly across different contexts. Research has primarily investigated the specific emotions students experience when receiving feedback and the correlation between these emotions and L2 achievement. These studies highlight that positive emotions (e.g., enjoyment) are strongly associated with higher achievement and deeper engagement, while persistent negative emotions (e.g., boredom) tend to adversely affect learning outcomes (Dewaele and Li, 2021; Tsang and Dewaele, 2023; Li and Zhang, 2026). In particular, existing scholarship has centered on instructor feedback, investigating how different feedback styles can foster positive emotions and increase pedagogical effectiveness (Mahfoodh, 2017; Han and Hyland, 2019; Geng and Yu, 2024). Other studies have explored learners’ emotional responses to alternative feedback sources, such as peer review and automated evaluation systems (Zhou et al., 2020; Yu et al., 2021). Collectively, these efforts illuminate the context-sensitive nature of feedback and the discrete emotions perceived by students across various instructional scenarios.

Over the past few years, the emergence of generative artificial intelligence (GAI) has revolutionized L2 writing instruction (Wang et al., 2024). Emerging GAI tools, such as ChatGPT, provide immediate evaluations of students’ grammar, usage, content, and organization, offering a promising alternative to traditional forms of feedback (Li and Collins, 2026). In this regard, pioneering studies have begun to explore GAI-assisted feedback within the domain of L2 learner engagement (Song and Song, 2023; Chen et al., 2024; Yeung, 2025), investigating the extent to which these tools support students’ cognitive, behavioral, and affective responses. Further research has documented the significant impact of integrated AI feedback on L2 students’ presentation performance (Sichterman et al., 2025), writing quality (Luo et al., 2025), and process-oriented revision (Helm and Hesse, 2026). However, while a growing body of research highlights the benefits of AI feedback in facilitating writing revision, existing research has primarily concentrated on its effectiveness and feasibility, leaving students’ emotional reactions to GAI-generated feedback and their subsequent self-regulatory behaviors an under-exploited realm. The integration of GAI into L2 education significantly shapes students’ academic development, in a way that their emotional responses can profoundly influence learning activities in these settings (Yang and Zhao, 2024). Given the complex interrelationship between learners’ academic emotions and their use of self-regulatory strategies in EFL learning (Shen et al., 2023), alongside the timely and dynamic nature of GAI-driven feedback (Banihashem et al., 2024), it can be posited that the emotions triggered by GAI may differ significantly from those elicited by traditional feedback formats. While previous studies have examined effectiveness and engagement, the emotional and regulatory dimensions of GAI-generated feedback, particularly in comparison to teacher feedback, remain under-explored. To address this potential query, the present study investigates the extent to which academic emotions triggered by GAI-supported feedback differ from those elicited by teacher feedback, as well as the corresponding strategies students employ to regulate these emotions in the context of argumentative writing.

## 2 Conceptual framework

### 2.1 Academic emotions in feedback on L2 writing

Academic emotions refer to a set of emotions experienced by learners during academic activities that are directly linked to learning, classroom instruction, and achievement (Pekrun et al., 2002). Existing research has primarily focused on the relationship between teacher feedback, L2 students’ emotional responses, and writing achievement. As feedback is a significant contributor to writing engagement, scholars have argued that positive teacher comments enhance students’ receptivity and facilitate proactive behaviors, whereas negative emotions typically impede revision and reflection (Pitt and Norton, 2016; Mahfoodh, 2017). Conversely, other studies have found that negative emotions, such as anxiety, do not consistently exert an adverse impact on students’ motivation and revision (Han and Hyland, 2019; Liu and Xin, 2025). However, less attention has been paid to the emotional experiences associated with receiving writing feedback from GAI tools during the revision process. Unlike traditional teacher feedback, GAI tools allow students to take the initiative in training, evaluating, and interpreting feedback based on their individualized expectations. This shift suggests that students may navigate complex cognitive and emotional dynamics while learning to write. As GAI tools are increasingly recognized by instructors and researchers as a valuable source of immediate feedback, a consensus is growing regarding their role as a complement to human feedback in writing instruction (Guo and Wang, 2024; Xu, 2025; Li and Collins, 2026). Nonetheless, empirical evidence concerning the emotions evoked by interacting with GAI tools during L2 writing revision remains limited. Specifically, GAI-generated feedback has not yet been fully integrated into the L2 English curricula of Chinese universities, which may lead to varied student perceptions and behaviors toward teacher versus GAI-generated feedback. Against this backdrop, the present study aims to fill this research gap by adopting a holistic perspective to compare the academic emotions experienced by L2 students when engaging with both feedback sources.

To align with our research objectives, we adopted the 2 × 2-dimensional taxonomy of academic emotions (Pekrun et al., 2002). This framework posits valence and activation as the two primary dimensions of academic emotions, serving as fundamental determinants for various emotional effects. While valence denotes the positive–negative spectrum of emotion, activation refers to the level of physiological arousal, encompassing both activating and deactivating states. Within this taxonomy, academic emotions are categorized into four distinct groups: positive activating (e.g., enjoyment, hope), positive deactivating (e.g., relief, relaxation), negative activating (e.g., anxiety, shame), and negative deactivating (e.g., boredom, hopelessness). Accounting for both valence and activation is essential for a comprehensive classification, as emotions with similar valence but different activation levels have been demonstrated to influence learning outcomes in distinct ways (Han and Hyland, 2019; Pekrun et al., 2002). In addition to valence and activation, Pekrun and Linnenbrink-Garcia (2012) introduced the concept of object focus to further explain the psychological functions of emotions. According to this criterion, academic emotions are further classified into achievement, epistemic, topic, and social emotions. Achievement emotions relate directly to academic activities and outcomes; epistemic emotions concern the cognitive and qualities of learning; topic emotions pertain to the content of learning materials; and social emotions involve interactions with others, such as teachers and peers. Specifically regarding achievement emotions, Pekrun (2006) identified three sub-factors: prospective outcome emotions, retrospective outcome emotions, and activity emotions, which correspond to anticipated outcomes, past outcomes, and the task at hand, respectively (Han and Hyland, 2019). This multi-dimensional taxonomy provides a robust analytical framework for capturing the complexity of emotions in academic environments. Furthermore, it is particularly relevant to the context of L2 writing feedback, as it underscores the importance of a nuanced understanding of learners’ emotional responses (Han and Hyland, 2019; Liu and Xin, 2025). Consequently, this integrated framework (Pekrun et al., 2002; Pekrun and Linnenbrink-Garcia, 2012) is well-suited for this study, enabling a broader investigation into feedback-aroused emotions across diverse scenarios.

### 2.2 Emotion-regulation strategies in feedback on L2 writing

Emotion regulation is defined as the process by which individuals influence the emotions they have when emotions arise, and how these emotions are experienced or expressed (Gross, 1998). This process involves utilizing various strategies that are, at least partially, conscious and goal-directed (Bielak and Mystkowska-Wiertelak, 2020). Originally conceptualized by Gross (1998, 2015), the process model of emotion regulation has been recognized as a well-acknowledged framework for analyzing emotion regulation within L2 learning and teaching contexts. These strategies are categorized according to the different stages of the emotion-generation process and are typically divided into five components: situation selection, situation modification, attentional deployment, cognitive change, and response modification. Specifically, situation selection involves selecting which situation an individual will be exposed to in anticipation of certain emotional responses. Situation modification refers to changing the relevant situational aspects to influence the person’s emotional impact. Attentional deployment concerns which situational aspects the person will perceive, either externally or internally, so as to alter their emotional responses. Cognitive change refers to the change in the person’s cognitive representation of a situation, with reappraisal being the most commonly examined type. Response modification concerns the direct adjustment of the physiological, experiential or behavioral aspects of the emotional responses.

Pekrun (2006) proposed the control-value theory of achievement emotions, postulating that emotions are primarily driven by control and value appraisals. This framework provides a lens to analyze the antecedents and effects of emotions experienced within achievement and academic contexts. Control appraisal refers to learners’ perceptions of their competence to control past, present, and future activities or outcomes, while value appraisal refers to learners’ evaluations about the value of activities or outcomes. Building upon these assumptions, Pekrun (2006) developed a four-dimensional taxonomy to identify the regulation of these emotions: emotion-oriented, appraisal-oriented, problem-oriented, and situation-oriented regulation. Specifically, emotion-oriented regulation directly addresses achievement emotions (e.g., by focusing or distracting attention); appraisal-oriented regulation addresses the control and value antecedents of emotions (e.g., by restructuring expectancies and attributions); problem-oriented regulation concentrates on improving academic learning and achievement underlying perceived control (e.g., by acquiring study skills); and situation-oriented regulation attempts to modify situational circumstances defining controllability and value (e.g., by asking for a reduction of task demands). When integrated with the process model of emotion regulation conceptualized by Gross (1998, 2015), these two models frame emotions, their antecedents, and their effects within a reciprocal pattern. This synthesized theoretical perspective is well-tailored for the present study, providing a powerful means to reveal the complexities of students’ emotion-regulation strategies in response to feedback.

In general, existing research on emotion regulation within the domain of L2 writing feedback has largely elucidated the link between students’ emotional experiences and their corresponding regulation strategies, highlighting the nuances and variations in regulatory practices. For instance, Geng and Yu (2024) identified task-related regulation, cognitive change, co-regulation, and attention deployment as the primary emotion-regulation strategies used by doctoral students to tame and smooth their emotions when receiving feedback on academic writing. In the context of Chinese as a Foreign Language (CFL), Liu and Xin (2025) examined the emotional responses and regulatory strategies of two students, finding that they utilized emotion-oriented, appraisal-oriented, and situation-oriented strategies to alleviate negative emotions. While existing research on L2 students’ emotion regulation has offered profound insights into its multifaceted nature during in teacher feedback situations, insufficient attention has been paid to the emotional experiences and strategies learners employ in AI-based contexts, particularly regarding comparative analyses between these two feedback modes. Among the limited empirical studies available, Yang and Zhao (2024) reported that Chinese EFL learners utilized various antecedent-focused and response-focused strategies to regulate AI-induced emotions. Furthermore, Li and Collins (2026) investigated students’ perceptions of instructor, peer, and AI-generated feedback, revealing a strong preference for human instructors over the other two sources. Additional empirical evidence is needed to uncover the mechanisms and taxonomies of L2 emotion regulation in AI-sourced feedback contexts. Given this, the present study aims to compare L2 students’ academic emotions and corresponding regulation strategies across teacher and GAI-generated feedback contexts by addressing the following two questions:

1. To what extent do L2 students’ academic emotional experiences in response to teacher feedback and GAI-generated feedback differ on argumentative writing?
2. How do these students differentially employ emotion-regulation strategies to regulate the emotions triggered by teacher and GAI-generated feedback?

## 3 Research methods

### 3.1 Context, participants, and instruments

This study was conducted in a 16-week postgraduate English course at a Chinese university, featuring two lectures per week. Argumentative writing instruction served as a core component of the curriculum and an essential means of preparing students for academic and dissertation writing. To collect rich and relevant data, a convenience sampling technique was employed for participant selection. Selection criteria included English proficiency, prior experience with GAI tools, and the frequency of receiving written assignments. Based on these criteria, 86 participants (37 males and 49 females, all Chinese nationals) were enrolled from two classes taught by the same instructor, who also served as the second researcher. All participants were first-semester accounting majors in a three-year academic program. They had achieved average scores of 70 and 68 (out of 100), respectively, on the National Postgraduate Entrance Examination (NPEE). As a high-stakes nationwide test taken by undergraduates seeking admission to postgraduate programs in China, these scores indicated that the participants possessed intermediate to upper-intermediate English proficiency. Prior to the commencement of the study, all participants were informed of the research objectives, assured of data confidentiality, and provided with written consent forms. To alleviate concerns regarding potential negative repercussions, participants were assured that their contributions would not affect their academic assessments for the course. This measure was intended to establish an open, secure, and honest atmosphere for sharing.

Furthermore, this study utilized a variety of GAI-based feedback tools during the revision process for two primary reasons. First, to capture authentic emotional responses in a naturalistic setting, we allowed participants to select tools based on their personal preferences and prior experience. Second, due to local telecommunication regulations and service availability, consistent access to a single international GAI application (such as ChatGPT) is restricted for many participants within the campus environment. Therefore, we granted participants the autonomy to use any accessible GAI tools (e.g., ChatGPT, Deepseek, Doubao or others). To maintain methodological rigor, we standardized the prompting instructions for all participants, ensuring the overall effectiveness and consistency of the GAI-supported revision activity.

**Figure 1:**
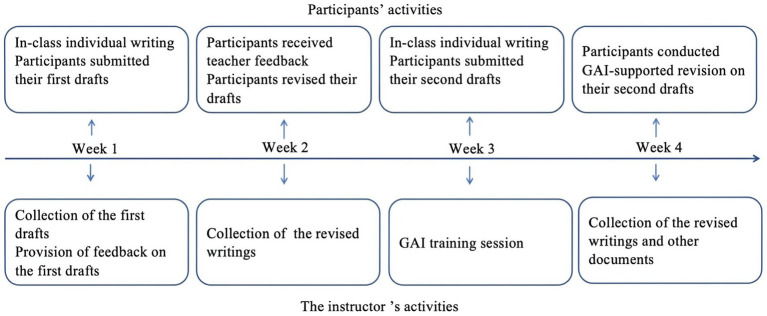
The procedure of two argumentative writing sessions.

To address the research questions effectively, the experimental procedure was designed to encompass two sequential feedback stages: teacher feedback followed by GAI-generated feedback. The primary rationale for this two-phase comparative design was to ensure that participants could gain an authentic and comprehensive understanding of the distinct characteristics of both feedback modes. By engaging in these two contexts successively through different argumentative writing tasks, participants were afforded a tangible and immediate basis for comparison. This approach aimed to elicit genuine emotional responses in real-world settings while allowing for the statistical analysis of differences in emotional and regulatory frequency across the two paired contexts. As depicted in Figure 1, the four-week study comprised two rounds of argumentative writing activities focused on distinct guided topics. In Week 1, the instructor tasked participants with completing an individual writing assignment within a single class period without the use of learning aids. Upon submission of these initial drafts, the instructor—an experienced writing teacher—reviewed, graded, and provided written corrective feedback based on the scoring criteria of the College English Test Band-6 (CET-6), a nationwide standardized test in China. In Week 2, participants received their graded essays and were required to rectify linguistic errors before resubmitting their revised drafts. In Week 3, participants completed a second argumentative writing task following the same in-class procedure as the first. For this session, however, they were instructed to retain their drafts for GAI-based self-directed revision after class. To facilitate this, the instructor dedicated one class period to training participants on effective prompting strategies, covering both technical skills and operational demonstrations. Specifically, the instructor guided participants in drafting standardized prompts aligned with CET-6 criteria for linguistic, expressive, and structural evaluations. Notably, participants were restricted to using GAI tools solely for generating revision suggestions rather than for complete rewriting. One week later, participants submitted their second initial drafts, the final revised versions, and screenshots documenting their dialogic revision process with the GAI tools.

### 3.2 Data collection

Adopting a qualitative study design, data for this research were collected from multiple sources: participants’ written retrospective reflections, their essays (including teacher feedback), screenshots of the GAI-assisted revision process, and semi-structured interviews. Regarding the retrospective reflections, participants completed a reflective journal of at least 500 Chinese characters within 40 min of class time following two argumentative writing sessions. These journals, titled “Reflections on the Two English Writing Feedback Activities”, prompted students to describe the emotional experiences they perceived during both the teacher feedback and GAI-generated feedback scenarios, specifically detailing how they processed and responded to these feelings. To ensure confidentiality, participants were permitted to submit their reflections anonymously. For the in-depth interviews, we categorized participants from both classes into high, medium, and low performance groups based on the top and bottom 25% of their grades on the first essay. Three participants were selected via voluntary participation from each of the three performance groups within each class, resulting in a total sample of 18 participants. These follow-up interviews served to supplement the written reflection data by clarifying ambiguities and capturing more explicit affective expressions. To encourage honesty and mitigate social desirability bias, the interviews were conducted by the first researcher who was not the course instructor, and were divided into two parts. First, the researcher encouraged participants to freely articulate the positive and negative feelings evoked by teacher and GAI-generated feedback, as well as their coping strategies for negative emotions. Furthermore, participants were asked to recall their prospective, concurrent, and retrospective emotional experiences and regulation strategies, with a specific emphasis on the differences between teacher and GAI-generated feedback across these stages. All interviews were conducted in Mandarin Chinese, audio-recorded, and transcribed for qualitative content analysis. Individual interviews lasted between 12 and 20 min, for a combined total of 283 min.

### 3.3 Data analysis

Qualitative content analysis was employed to systematically examine the integrated data (Miles et al., 2014). The coding process followed two stages:labeling discrete academic emotions and identifying emotion-regulation strategies based on established frameworks (Pekrun and Linnenbrink-Garcia, 2012; Gross, 2015). To enhance analytical rigor and support the qualitative claims regarding differences between the two contexts, a quantitative comparison was also conducted. As the analysis involved comparing emotion frequency counts from the same participants across two feedback stages, Wilcoxon signed-rank tests (Siegel and Castellan, 1988; Field, 2013) were performed using SPSS 26.0 to determine whether the differences in reported positive and negative emotions between teacher and GAI feedback were statistically significant.

Drawing on participants’ self-reported journals, the first round of coding involved a semantic analysis of the written reports to extract emotions. These were then deductively classified according to the taxonomy of academic emotions framework proposed by Pekrun and Linnenbrink-Garcia (2012). We also tallied the frequency of each emotion type; for instance, “hope” was coded as a positive and activating emotion in terms of valence and activation and was identified as a frequently occurring emotion related to object focus. Similarly, emotion-regulation strategies were initially coded through a detailed review of the reflective journals, referencing the regulation models proposed by Gross (1998, 2015) and Pekrun (2006). Expressions reflecting the strategies participants employed to address feedback-aroused emotions were identified and categorized into various dimensions. Concurrently, we recorded the occurrences of these strategies and noted the specific emotions being regulated. For example, “anxiety,” the most frequently mentioned negative emotion, was identified as being managed through strategies such as situation selection, attentional deployment, cognitive change, and response modification. The second round of coding followed a similar procedure, deductively labeling academic emotions and regulation strategies by analyzing the transcribed interview data. Finally, we cross-referenced the coded data for the 18 interviewed participants to eliminate any redundant counting of emotions or strategies between their written reports and interview transcripts.

To minimize subjective bias and distorted interpretations during the coding process, two researchers independently coded the reflective journals and interview transcripts before cross-comparing their results. For instance, the excerpt, “The responses from GAI deviated far from my intended meaning, making me feel stressed and uncontrollable,” was categorized as “anxiety” (a negative activating emotion), while “Insufficient proficiency to implement the teacher’s suggestions” was coded as “hopelessness” (a negative deactivating emotion). These distinctions were made because the activation level of negative emotions appeared to have varying effects on participants’ intrinsic motivation to proceed with revisions. For these participants, anxiety could either impair or facilitate the use of regulatory strategies, whereas hopelessness was purely detrimental as it eroded their motivation. To ensure the agreement among the two researchers, a coding reliability analysis (Cohen, 1960) using the Kappa statistic was performed in SPSS 26.0. The results yielded a Kappa value of 0.84 for academic emotions and 0.81 for emotion-regulation strategies, both of which were higher than 0.7, thus claimed a good level of agreement according to Landis and Koch (1977). Any remaining discrepancies were resolved through iterative discussions until a consensus was reached on a unified coding decision.

**Figure 2:**
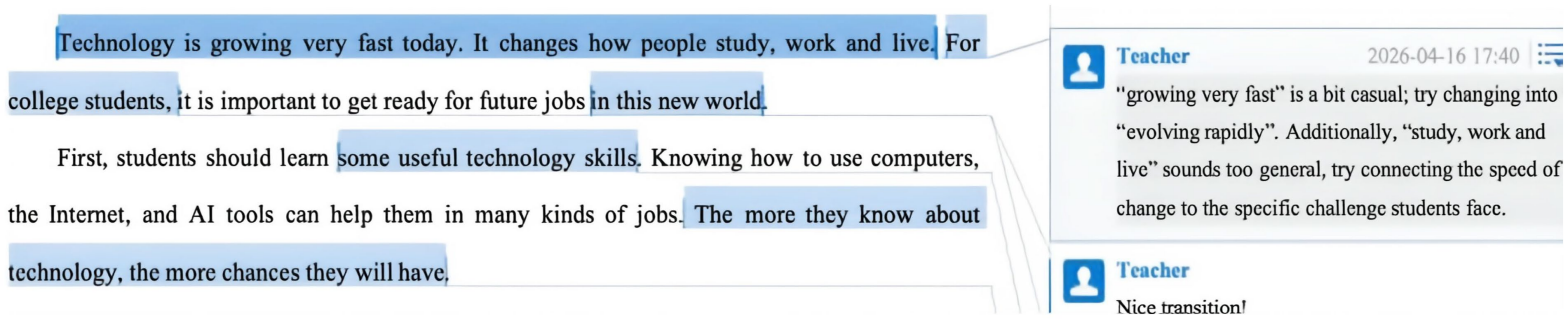
Sample of teacher feedback.

**Figure 3:**
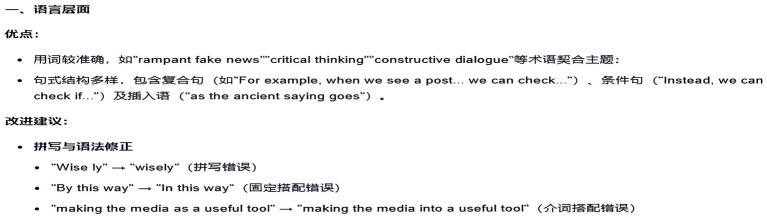
Sample of GAI-generated feedback.

We also utilized multiple complementary data sources, including participants’ essays with teacher feedback and screenshots of the dialogic GAI revision process. All above data were sorted out and archived by each participant, followed by horizontal comparisons across different sources of data. Specifically, participants’ self-reported emotions in reflective journals and interviews were cross-referenced with their essays containing teacher feedback (see Figure 2) and screenshots of GAI interactions (see Figure 3). This step was employed to confirm the consistency between participants’ self-reported reflections and their actual revision behaviors. As illustrated in Figure 2, teacher feedback was characterized by localized and handwritten annotations focusing on specific linguistic errors and content logic. Conversely, Figure 3 displays a typical GAI-generated feedback session, featuring structured and multi-dimensional comments delivered in an immediate, conversational interface. During the coding process, these visual data sources served as a critical evidence check. For instance, when participants reported “anxiety” regarding GAI’s overloaded suggestions, the researchers referred back to the GAI screenshots to verify the volume and complexity of the feedback provided. This triangulated approach helped to better understand the relationship between teacher written feedback, GAI-generated feedback, students’ emotional responses, and the success of their revisions.

## 4 Findings

### 4.1 Academic emotions perceived by L2 students in response to teacher and GAI-generated feedback

The integration of qualitative coding and statistical analysis revealed distinct emotional profiles across the two feedback contexts, categorized according to the three-dimensional taxonomy of academic emotions (Pekrun et al., 2002; Pekrun and Linnenbrink-Garcia, 2012). Overall, twenty-three academic emotions were identified among L2 students during the process of receiving teacher and GAI-generated feedback. These emotions were classified into four categories: achievement, epistemic, topic, and social emotions. The specific academic emotions and their respective frequencies are presented in Table 1.

**Table 1:** L2 students’ perceived academic emotions in teacher and GAI-generated feedback.

Object focus	Positive		Negative	
	Activating	Deactivating	Activating	Deactivating
Activity	Hope (34/4) enjoyment (20/4) inspiration (8/5)	Contentment (12/21) awe (17/0) tranquility (5/8)	Anxiety (18/29) shame (0/2)	Disappointment (1/3) hopelessness (1/2)
Prospective outcome	Hope (11/32) curiosity (3/5)	–	Nervousness (5/21) fear (2/0)	–
Retrospective outcome	–	Contentment (9/5)	Fear (0/7)	Dissatisfaction (0/3)
Epistemic emotions	Enjoyment (17/0) interest (4/7) thrill (0/13) surprise (0/25) hope (0/13)	–	Frustration (9/26) anxiety (2/14) confusion (7/17)	Disappointment (0/14)
Topic emotions	Interest (2/1)	–	–	–
Social emotions	Trust (26/0) empathy (15/6) gratitude (7/0) admiration (2/0)	–	Shame (9/0)	–

The numbers in parentheses before and after the “/” represent occurrences of the emotion experienced separately in teacher feedback and GAI-generated feedback.

As illustrated in Table 1, achievement emotions, comprising activity, prospective, and retrospective outcomes, emerged as the most frequently reported category of academic emotions. In contrast, only one emotion type, interest, was identified within the topic emotions category. Statistical analysis revealed significant differences in the distribution of emotional valence between the two conditions. Positive emotions (192/149), notably hope and enjoyment, were non-significantly more prevalent in teacher feedback (*M* = 4.57) compared to GAI-generated feedback (*M* = 3.61; *Z* = 1.901, *p = 0.061 >* 0.05). On the contrary, negative emotions (52/138), such as anxiety and frustration, showed a significantly higher occurrence in the GAI-generated feedback setting (*M* = 3.22) than in the teacher feedback context (*M* = 1.13; *Z* = -2.132, *p = 0.036* < 0.05). These statistical results provide evidence that teacher feedback elicited more favorable emotional responses, whereas GAI feedback tended to induce greater distress.

Among all coded emotions, positive activating emotions were the most prevalent in terms of both frequency and diversity. Specifically, hope (45), enjoyment (37), and trust (26) were the three positive activating emotions most frequently triggered by teacher feedback. Most participants noted that the teacher’s comments were clear and accurate, prompting them to take immediate action to revise their work. Additionally, they reported that the teacher’s comments accurately highlighted cognitive and linguistic weaknesses they had previously overlooked. This made the feedback feel highly insightful, as reflected in excerpts like, “hoped to address all errors underlined by the teacher” (S41, reflective journal) and “hoped to make progress” (S6, reflective journal). Enjoyment was also a prominent achievement emotion, as evidenced by comments like, “the teacher’s comments made me feel confident and pleasant” (S55, interview). Participants further expressed a sense of trust upon receiving teacher feedback by stating “trusting teacher’s professional experience” (S65, reflective journal) and “I believe in the teacher’s comments because she knows me well” (S8, interview). In contrast, hope (49), surprise (25), and thrill (13) were the positive activating emotions most frequently declared by participants in response to GAI-generated feedback. Hope, which was often linked to prospective outcomes, was reported 32 times in the GAI context. Participants expressed their anticipation before receiving the results, noting they “expected for GAI’s feedback” (S4, reflective journal). They also emphasized their expectancy when interacting with GAI tools, stating they “hoped that AI would provide precise feedback tailored to my instructions” (S24, reflective journal). Surprise was coded as an epistemic emotion because it stemmed from the students’ cognitive activity during revision, as participants confessed that “GAI offered me many unexpected fresh ideas” (S11, reflective journal) and “surprisingly inspired by its (ChatGPT’s) perspectives” (S52, reflective journal). Finally, the emotion of thrill emerged when participants interacted with the technology, as seen in comments like, “When the chatbox was constantly flooded with a lot of fresh insights for my writing, I was thrilled” (S29, interview). These findings highlight that the immediate provision of content-rich feedback by GAI tools evokes positive emotions and boosts participants’ motivation to engage in learning.

Regarding positive deactivating emotions triggered by teacher feedback, the most prevalent were contentment (21) and awe (17). Participants expressed their sense of contentment by noting, “I was contented with my improvement in recognizing my limitations” (S9, reflective journal) and “I have acquired new insights and skills in argumentative writing” (S41, reflective journal). Some participants also felt a sense of awe toward the teacher’s feedback. One student stated frankly that the teacher was perceived “more like an authority who could not be questioned” (S37, interview). Another admitted, “I didn’t approach the teacher even though I was confused by her comments, as this would put me under too much pressure” (S56, interview). Similarly, contentment (26) was the predominant emotion experienced in the GAI-based context. Participants reported feeling this way when they continuously sought feedback from GAI tools to enhance their learning engagement. For instance, one student shared, “As my interaction with the tool progressed, it brought me more contentment” (S3, reflective journal), while another noted, “I fully approved of its efficiency and accuracy in language proofreading” (18, reflective journal).

In respect of negative activating emotions, GAI-generated feedback evoked a greater frequency and variety than teacher feedback. Anxiety was the most prevalent emotion across both feedback conditions (20/43). Some participants noted that “I felt a surge of anxiety when I saw numerous corrections on fundamental errors my teacher had made in the draft” (S2, reflective journal) and “The responses from GAI deviated significantly from my intended meaning, making me feel stressed and out of control” (S24, reflective journal). Frustration (9/26) was also prominently reported, particularly as an epistemic emotion within GAI-based settings. For instance, one participant expressed frustration because “the suggested words and expressions were well above my English level, making it impossible for me to apply them” (S63, interview). In contrast, participants were more likely to report feelings of shame (9/2) in the teacher feedback scenario, primarily due to the worry of “exposing shortcomings in front of the teacher and classmates” (S10, reflective journal). Additionally, nervousness (5/21) was more common in the GAI-based context, often occurring as students anticipated agents’ response. This was reflected in comments such as, “I felt extremely nervous when submitting my draft to the app” (S15, reflective journal) and “*I opened Doubao* (*a GAI language model developed by ByteDance in China*) *with a mixed feeling of expectancy and nervousness*” (S34, reflective journal). Finally, confusion (7/17) and fear (2/7) were also notable. One student stated that “the suggestions from the tool concerning logical structure were too obscure for me to implement” (S7, interview), while another admitted, “I completely relied on AI to write for me… I feared that over-reliance on it could undermine my English proficiency” (S32, interview).

Regarding negative deactivating emotions, some participants experienced feelings of disappointment (1/17) and hopelessness (1/2) during the revision process. This was reflected in comments such as: “Some suggestions from GAI were too complicated and formulaic to be integrated into my essay. In contrast to my original draft, the GAI-revised version felt impersonal and lacked a human touch” (S6, reflective journal), and “Though the resubmitted writing looked good, I know it was the machine that replaced my own voice. I learned nothing” (S14, interview).

### 4.2 Emotion-regulation strategies employed by L2 students in teacher and GAI-generated feedback contexts

The results of the mixed-methods analysis revealed that participants employed 16 specific strategies to regulate their academic emotions. Quantitative comparisons indicated that participants used emotion-regulation strategies (107/196) significantly more frequently in the GAI-generated feedback sessions (*M* = 4.78) than in the teacher feedback sessions (*M* = 2.48; *Z* = -2.277, *p* = 0.025 < 0.05). This suggests that the GAI-mediated context necessitated more active and complex emotional regualtion. Drawing on Gross’s (1998, 2015) model, the regulatory strategies used by participants were closely associated with the activation levels of the emotions they experienced. These strategies were categorized into five groups—situation selection, situation modification, attentional deployment, cognitive change, and response modification, which collectively encompassed emotion-oriented, appraisal-oriented, problem-oriented and situation-oriented regulation. Table 2 presents the specific strategies adopted by L2 students, their frequency of occurrence, and the academic emotions being regulated.

**Table 2:** Emotion-regulation strategies adopted by L2 students in teacher and GAI-generated feedback sessions and examples of academic emotions being regulated.

Categories	Specific strategies	Academic emotions being regulated
Situation selection (5/11)	Situation access (2/1)	Anxiety shame frustration
	Situation avoidance (3/10)	
Situation modulation (59/76)	Turning to teacher for assistance (4/0)	Curiosity hope frustration confusion fear
	Comparing with peers’ performance (8/0)	
	GAI tool replacement (0/4)	
	Improving writing competence (40/59)	
	Reflecting on shortcomings (7/13)	
Attention deployment (12/25)	Attention distraction (4/9)	Anxiety shame frustration confusion interest
	Attention reinforcement (6/5)	
	Attention selection (2/11)	
Cognitive change (23/75)	Enhancing control appraisal (14/43)	Anxiety confusion frustration fear disappointment
	Decreasing control appraisal (6/11)	
	Increasing value appraisal (3/21)	
Response modulation (8/9)	Expression suppression (3/0)	Anxiety frustration confusion
	Venting feelings with peers (5/7)	
	Talking with significant others (0/2)	

The numbers in parentheses before and after the “/” represent occurrences of the sub-strategy employed separately in teacher feedback and GAI-generated feedback.

To begin, situational modification (59/76) emerged as the most frequently employed strategy, encompassing the broadest range of sub-strategies aimed at modifying both intrinsic and extrinsic situations. Data analysis revealed that several participants experienced anxiety regarding criticisms and corrections upon receiving teacher feedback. Even after reviewing the corrective comments, many remained confused about how to implement accurate revisions. To manage these emotions, students utilized the sub-strategy of seeking the teacher’s assistance (4/0) to mitigate anxiety and resolve confusion; as one student noted: “For some unclear suggestions, I asked the teacher for additional explanations” (S72, reflective journal). Another sub-strategy applied exclusively in the teacher feedback scenario was comparing with peers’ performance (8/0). This tactic involved students inquiring about their peers’ comments to reevaluate their own competence and, to some extent, satisfy their curiosity. This sub-strategy helped regulate negative emotions such as anxiety.

#### 4.2.1 Extract 1

*I could not help but glance at the essays of the classmates sitting beside me to see what comments the teacher had given them. I was so anxious and curious, wanting to know if they were struggling as much as I was. It was comforting to realize I was not the only one facing extensive revisions* (S9, interview).

In addition, the third sub-strategy identified was GAI tool replacement (0/4). This occurred when participants felt frustrated by various barriers encountered during GAI-based revision. To overcome these obstacles, they transitioned to alternative GAI tools to obtain the expected feedback. These three sub-strategies were employed by participants to mitigate negative activating emotions by modifying extrinsic situations. Conversely, two sub-strategies were adopted to modify participants’ intrinsic situations: improving writing competence (40/59) and reflecting on shortcomings (7/13), with the former being the most frequently utilized sub-strategy in this study. In both teacher and GAI-generated feedback scenarios, participants reported alleviating negative emotions (e.g., anxiety and confusion) by carefully reviewing and analyzing comments, thereby proactively engaging in the revision process. As their focus shifted toward error rectification, their negative activating emotions subsided. As two students noted, “I implemented the teacher’s suggestions point by point, addressing all spelling and grammar errors” (S66, reflective journal), and “I classified errors by types—spelling, grammar, collocation—to facilitate further learning” (S43, reflective journal). Furthermore, some participants reflected on their weaknesses and internalized new writing skills during the revision process, stating, “I sorted out all errors in a correction notebook, including spelling mistakes and vocabulary misuse” (S57, reflective journal). Another participant looked toward future improvement, remarking in an interview, “To leverage GAI more efficiently, I plan to master the art of prompting, ensuring I elicit more constructive feedback” (S12).

Cognitive change (23/75) was the second most commonly used strategy, comprising enhancing control appraisal (14/43), decreasing control appraisal (6/11), and increasing value appraisal (3/21). Among these, enhancing control appraisal was the most frequently adopted. This approach was utilized when participants sought to alter perceptions of their ability to complete revision tasks. Although participants viewed unsuitable output from GAI tools as a major barrier to revising their writing—experiencing negative activating and deactivating emotions such as disappointment, anxiety, confusion, and frustration, they also highlighted the extra time and effort required to evaluate GAI-generated feedback. The employment of enhancing control appraisal facilitated a positive emotional transformation, encouraging participants to adopt a constructive perspective toward negative comments and to invest greater effort in their learning engagement.

#### 4.2.2 Extract 2

*Initially, I felt disappointed in myself, wondering how I could have made so many mistakes. However, I soon shifted my perspective, choosing to channel that disappointment into motivation for revision. After all, identifying a problem is the first step toward solving it* (S26, interview).

#### 4.2.3 Extract 3

*When confronted with a barrage of critical feedback from GAI, I was initially astonished and overwhelmed. However, I regained my composure and maintained an open mind, beginning to view myself as the ‘decision-maker’ and GAI as a ‘consultant’ tasked with offering alternative perspectives* (S11, interview).

Decreasing control appraisal was utilized when participants, discouraged by language deficiencies, sought to relieve discomfort by accepting their limitations. For instance, one student noted: “I made a real effort to revise my writing based on the teacher’s suggestions, but it was really challenging and far beyond my current level” (S64, interview). Similarly, increasing value appraisal was adopted as participants regulated negative emotions by acknowledging the importance of revision for improving their writing skills. As one student shared: “I reminded myself to focus on the root causes of my poor performance to prevent these errors from happening next time” (S8, reflective journal). Additionally, participants noted that the timely and dialogic feedback provided by GAI tools helped mitigate anxiety and confusion. One student reflected: “As I input more precise prompts into GAI, it provided responses that aligned with my expectations. This filled me with genuine satisfaction throughout the rest of the revision process” (S12, reflective journal). Furthermore, participants placed a higher value appraisal on GAI-generated feedback when they exerted greater agency in working with the tools rather than uncritically accepting responses. This shift is reflected in comments such as: “As I reflected deeply instead of passively accepting the tool’s responses, the revision practice proved to be quite rewarding” (S5, reflective journal).

The attention deployment strategy (12/25), encompassing attention distraction (4/9), attention reinforcement (6/5), and attention selection (2/11), was reported less frequently in this study. Participants employed attention distraction to redirect their focus away from the writing task, thereby avoiding more intense negative emotions. For instance, upon receiving lengthy feedback from GAI tools, participants diverted their attention to temporarily disengage from their emotional frustration.

#### 4.2.4 Extract 4

*A large number of corrective comments came as a blow to me, so I put them aside to cool off. I could not face such a daunting challenge at that moment, so I shifted my focus to other assignments instead. After a day or so, I made up my mind and sat down with a clear head to handle the revision* (S57, interview).

In contrast, attention reinforcement is a regulatory sub-strategy used by participants to address unpleasant feelings or potential difficulties by intensifying their focus on the learning activity. This is exemplified by one student who noted: “After carefully reading the teacher’s feedback, I immediately began revising—I act quickly, as this has always been my learning habit” (S18, interview). Attention selection, on the other hand, was primarily employed to cope with confusing or overwhelming feedback that triggered GAI-induced negative emotions. By using this strategy, participants engaged critically with suggestions to incorporate only the most helpful and relevant feedback suited to their needs.

#### 4.2.5 Extract 5

*Since some of the advanced vocabulary and complex grammatical structures were beyond my current proficiency, I selectively adopted suggestions that aligned with my level, rather than blindly pursuing complicated expressions or sophisticated wording* (S45, reflective journal).

The response modulation strategy (8/9) involves expression suppression (3/0), venting feelings with peers (5/7), and talking with significant others (0/2). To relieve negative emotions and restore emotional stability, participants frequently externalized their feelings. For example, one student remarked: “I talked to my classmates and said ‘Hey, how’s your writing going? Mine is so hard…’. It made me feel like we were in the same boat, and this gradually calmed me down” (S43, interview). Similarly, another noted: “I opened up about my frustrations with my brother who is a PhD. After reading my essay, he pointed out my problems and shared his experience” (S3, interview). In contrast, some participants chose to internalize their emotions, as one student noted: “unpleasant feelings could be addressed internally by myself” (S7, reflective journal). The strategy of situation selection was the least frequently used in this study, comprising situation access and situation avoidance. Situation access involves preparing psychologically and behaviorally before reading feedback, such as “choosing to revise in the library” (S12, interview). Situation avoidance was reported more often in the GAI-based context, referring to participants’ spontaneous reactions to GAI-generated feedback that triggered feelings of sensitivity and vulnerability. As one student stated: “Because the tool made the original sentences so complicated that I felt a sense of resistance and worried about losing my own style” (S8, reflective journal).

## 5 Discussion

This study compared the academic emotions and emotion-regulation strategies of 86 Chinese master’s students in teacher-led versus GAI-generated feedback contexts. A total of 23 discrete academic emotions were identified. These emotions were categorized by object focus (achievement, epistemic, topic, and social emotions) and classified into four types based on valence and activation, further extending the applicability of the framework developed by Pekrun and Linnenbrink-Garcia (2012) for analyzing academic emotions in diverse L2 learning scenarios. Comparative analysis revealed that Chinese L2 students experienced a dynamic range of positive and negative academic emotions before, upon and after receiving feedback in both settings. These results align with previous findings regarding the richness and complexity of L2 students’ academic emotions triggered by both teacher feedback and AI-based practices (Han and Hyland, 2019; Geng and Yu, 2024; Yang and Zhao, 2024). Specifically, hope, enjoyment, trust, surprise, and contentment emerged as the most frequent positive emotions. Conversely, anxiety, nervousness, confusion, and frustration were the most prevalent negative emotions. While most of these emotions were reported across both feedback situations, their relative prevalence varied.

A key contribution of this study is the discovery that GAI feedback causes an object focus shift within L2 writing. In traditional settings, the teacher-student dyad makes feedback a social-relational object. However, GAI feedback shifted the object focus from social interaction to the achievement process itself. This was proven in the fact that emotions such as awe, gratitude and trust were reported to be exclusive to the teacher feedback situation, whereas surprise, excitement and dissatisfaction were the typical characteristic of the GAI-generated feedback context. Furthermore, while teacher feedback elicited academic emotions marked by positive valence and high activation, GAI-generated feedback frequently triggered negative-activating emotions, most notably anxiety and frustration. This suggests that without the social support of a teacher, students feel a heightened responsibility for the final product, leading to “feedback-overload anxiety” (Yang and Zhao, 2024). These findings provided further evidence on the enduring value of teacher feedback in writing instruction. Qualitative analysis revealed that students perceived tailored teacher feedback as the most reliable and authoritative source, playing a critical role in helping them address key aspects of their writing. Positive emotions, including gratitude for a teacher’s dedication and the empathetic resonance of encouraging comments, bolstered students’ motivation for agentic learning. These results echo the belief that teacher feedback helps to affirm students’ individual identities and needs, thereby supporting their learning (Pitt and Norton, 2016; Mahfoodh, 2017; Li and Collins, 2026). However, the results also showed that the sense of awe students felt toward their teachers could occasionally hinder them from seeking further clarification, potentially undermining the feedback’s benefits. Unlike the social-relational focus of teacher feedback, GAI was viewed as an “impersonal tool,” shifting the emotional and cognitive burden to the student’s own ability to discern and verify information.

This study contributes to existing research by unveiling that while teacher feedback primarily triggers activity-related emotions in L2 students, GAI-generated feedback elicits a significantly higher frequency of epistemic emotions. This finding reveals a meaningful association between academic emotions, feedback turns, and students’ cognitive efforts. In the teacher feedback context, limited direct interaction led students to report emotions primarily triggered before the revision activity, which then remained relatively stable. Conversely, in GAI-mediated scenarios, although L2 students initially grappled with anxiety and other negative feelings during the stage of emotional arousal, their subsequent revision efforts facilitated a gradual shift toward reduced negative affect and increased positive emotions, such as excitement, surprise, and hope. It appears that the novel perspectives offered by GAI tools can promote students’ feedback-seeking behaviors; the more cognitive effort students invest in seeking dialogic feedback, the more likely they are to endorse the usefulness of these tools. This aligns with prior research suggesting that AI-induced emotions are less about the “result” and more about the “process” of negotiation between human and machine (Wang et al., 2024). Crucially, this study highlights students’ inconsistent responses to error revision when experiencing negative emotions triggered by GAI feedback. Students suffering from exacerbated negative deactivating emotions, such as disappointment and hopelessness, were less likely to exercise agency in seeking further feedback—a tendency partly attributable to low GAI-based feedback literacy and inherent technological biases. In contrast, and consistent with previous studies (Han and Hyland, 2019; Geng and Yu, 2024; Liu and Xin, 2025), students’ negative activating emotions, such as anxiety, frustration, and confusion, did not necessarily discourage them from improving their revisions; rather, these emotions could be transformed into positive drivers when appropriate strategies were employed to address them.

Furthermore, this study confirms that master’s students utilized five emotion-regulation strategies, encompassing 16 sub-strategies, to manage their emotions across two revision sessions. This finding provides further evidence for Gross’s (1998, 2015) process model of emotion regulation and align with existing research on the strategies L2 students employ to navigate negative emotions in feedback contexts (Han and Hyland, 2019; Liu and Yu, 2022). Despite the initial prevalence of negative emotions regarding GAI-generated feedback before and during the early stages of revision, students significantly increased the frequency with which they employed strategies to manage these unfavorable feelings. Among these strategies, situation modification was the most frequently reported, supporting the taxonomy proposed by Harley et al. (2019), which distinguishes internal situation modification from external situation modification. In the current study, internal situation modification occurred as students improved their intrinsic writing skills and reflected on their weaknesses to alleviate negative emotions (e.g., anxiety, frustration, and confusion) in both teacher and GAI-based feedback settings. Meanwhile, external situation modification was observed when students sought clarification for ambiguous feedback from the teacher, refined their prompts to generate better GAI outputs, or switched to different GAI tools for more accurate and precise suggestions.

The study also revealed that, in response to high-activation negative emotions (e.g., anxiety) induced by both teacher and GAI-generated feedback, students predominantly employed cognitive change strategies. The significantly higher frequency of cognitive change in the GAI setting suggests that L2 learners are not passive recipients of AI feedback; instead, they utilize regulation strategies to bridge the ‘control gap’ inherent in GAI interactions. As the findings suggest, students adopted the tactic of enhancing control appraisals to critically reassess received comments, evaluate their weaknesses objectively, and increase their perceived control over the effectiveness of their revisions. They mitigated unpleasant emotions by reframing the revision process as an opportunity for skill development rather than a definitive judgment of their inherent competence. Furthermore, this study adds a new dimension to the “enhancing control appraisal” tactic within the context of GAI-supported revision by discovering that students often engaged more deeply with GAI tools to address concerns regarding the detrimental effects of over-reliance. They sought to bolster their identities as proactive learners, viewing GAI-supported revision as a learning journey rather than a mere exercise in error correction. Concurrently, the attention selection sub-strategy was adopted to achieve these aims. For instance, students exercised their ability to engage in effective revision by selectively accepting relevant suggestions while rejecting redundant feedback. This aligns with the findings of Chen et al. (2024), who noted that L2 students often contested or ignored overwhelming and unclear feedback from ChatGPT in their argumentative essays. In contrast, the employment of the decreasing control appraisal strategy illustrated that students’ perceptions of their own linguistic deficiencies and low digital feedback literacy led them to lower their outcome expectancies. This, in turn, hindered them from exerting the effort necessary for effective revision. These findings imply that an L2 student’s preference for either enhancing or decreasing control appraisals is closely associated with their feedback literacy and learning mindset. This research extends previous work (Xu, 2025) by highlighting that emotion regulation is an essential component of AI feedback literacy. Learners who can effectively reappraise GAI outputs through a growth mindset are more likely to transition from negative-activating states to positive-activating states, thereby facilitating deeper and more meaningful revision.

## 6 Conclusions, implications, and limitations

This study examines master’s students’ academic emotions and their corresponding emotion-regulation strategies in response to teacher and GAI-generated feedback on argumentative writing. It identifies 23 distinct emotions (categorized by valence, activation, and object focus) and 16 emotion-regulation sub-strategies across five categories. By incorporating L2 students’ discrete academic emotions and their evolving responses to GAI feedback, this research bridges a significant gap and confirms the highly context-dependent, nuanced, and dynamic nature of academic emotions (Pekrun, 2006; Pekrun and Linnenbrink-Garcia, 2012; Harley et al., 2019). The findings reveal that while teacher feedback elicits a blend of positive and negative emotions, notably social emotions such as gratitude, trust, and awe, GAI-generated feedback initially triggered a prevalence of negative emotions, including anxiety, frustration, and confusion, due to barriers in accessing useful input. However, by employing effective strategies—primarily situation modification and cognitive change—students experienced an emotional reversal that ultimately enhanced their creativity and confidence in GAI-assisted learning.

By comparison, while teacher feedback excels at affirming students’ individual identities and ensuring high feedback uptake rates, GAI-supported revision offers distinct advantages in speed, convenience, and novelty. Given these complementary strengths, we recommend a hybrid feedback strategy that integrates GAI with teacher feedback to optimize L2 learners’ writing development. In this model, teachers should serve as the primary providers of personalized, guidance-oriented corrections, supporting students through multi-modal feedback—such as negotiable written and oral dialogic feedback. Regarding GAI-based feedback, teachers can guide students in making reflective comparisons between their own revised drafts and the GAI’s output, focusing on lexical accuracy, syntactic structure, cultural nuances, and other linguistic factors. Furthermore, an L2 student’s ability to regulate emotions is crucial for fostering resilience. This study highlights that teachers can address students’ emotional needs by tailoring their comments and preparing learners to navigate negative feelings in challenging learning contexts. Similarly, students should shift their focus from immediate emotional reactions toward overarching writing goals, adopting a more open-minded and cognitively mature approach to revision. Finally, as the findings reveal uneven technological feedback literacy among Chinese L2 students, which may cause those with lower literacy levels to experience more negative emotions when seeking feedback. Therefore, targeted feedback literacy training is essential to help students become more proficient within GAI-assisted educational environments.

While this study advances our understanding of academic emotions and their regulation within teacher and GAI-generated feedback contexts, several limitations warrant acknowledgment. First, constrained by the course curriculum, the study spanned only 4 weeks and comprised two writing sessions; it is possible that the findings would differ over a more extended experimental period. Future research should adopt a longitudinal approach to examine L2 learners’ emotional trajectories and anticipated actions, providing deeper insight into how perceptions of GAI-generated feedback evolve over time. Second, the participants were a homogeneous group of Chinese postgraduate accounting students with intermediate to upper-intermediate English proficiency. This homogeneity may obscure potential variations in emotional experiences and regulation strategies across different disciplines and proficiency levels. Future studies should involve more diverse samples to enhance the representativeness and generalizability of the results. Third, although reflections were submitted anonymously, the reliance on retrospective self-reporting may introduce bias, potentially undermining methodological rigor. Future research could incorporate observational methods to capture students’ instinctive emotional reactions and revision behaviors, thereby strengthening the triangulation of findings. Finally, the specific GAI tools used were not controlled in the current study. Future research should standardize the GAI feedback process to minimize variability in feedback quality, which could otherwise confound students’ emotional responses.

## Data Availability

The raw data supporting the conclusions of this article will be made available by the authors, without undue reservation.
